# Automated growth rate determination in high-throughput microbioreactor systems

**DOI:** 10.1186/s13104-017-2945-6

**Published:** 2017-11-25

**Authors:** Johannes Hemmerich, Wolfgang Wiechert, Marco Oldiges

**Affiliations:** 1 Institute of Bio- and Geosciences-IBG-1: Biotechnology, Forschungszentrum Jülich, Jülich, Germany; 2 Computational Systems Biotechnology (AVT.CSB), RWTH Aachen, Aachen, Germany; 3 Institute for Biotechnology, RWTH Aachen, Aachen, Germany; 4 Bioeconomy Science Center (BioSC), c/o Forschungszentrum Jülich, Jülich, Germany

**Keywords:** Microbioreactor, Online biomass monitoring, Growth rate, Exponential growth model, Quantitative microbial phenotyping

## Abstract

**Objective:**

The calculation of growth rates provides basic metric for biological fitness and is standard task when using microbioreactors (MBRs) in microbial phenotyping. MBRs easily produce huge data at high frequency from parallelized high-throughput cultivations with online monitoring of biomass formation at high temporal resolution. Resulting high-density data need to be processed efficiently to accelerate experimental throughput.

**Results:**

A MATLAB code is presented that detects the exponential growth phase from multiple microbial cultivations in an iterative procedure based on several criteria, according to the model of exponential growth. These were obtained with *Corynebacterium glutamicum* showing single exponential growth phase and *Escherichia coli* exhibiting diauxic growth with exponential phase followed by retarded growth. The procedure reproducibly detects the correct biomass data subset for growth rate calculation. The procedure was applied on data set detached from growth phenotyping of library of genome reduced *C. glutamicum* strains and results agree with previously reported results where manual effort was needed to pre-process the data. Thus, the automated and standardized method enables a fair comparison of strain mutants for biological fitness evaluation. The code is easily parallelized and greatly facilitates experimental throughout in biological fitness testing from strain screenings conducted with MBR systems.

**Electronic supplementary material:**

The online version of this article (10.1186/s13104-017-2945-6) contains supplementary material, which is available to authorized users.

## Introduction

Quantitative phenotyping of microbial strain libraries describes the assignment of performance indicators to each library item, i.e. strain, screened. The growth rate is an important performance indicator, because it is a suitable metric for biological fitness of microbial mutant strains [1]. For screening of mutant libraries after strain mutagenesis, parallelized and easy-to-handle cultivation systems are needed to realize sufficient experimental throughput. In this context, several microbioreactor (MBR) systems are available with integrated quasi-continuous biomass measurement based on optical density “BioScreen C” [2], image scanning “Growth profiler” [3] or backscatter (BS) signal “BioLector” [4].

Typically, parallel high-throughput growth experiments in MBR involve strain mutants which usually show deviating growth behavior like different lag-phases, final biomass concentrations, maximum growth rates or even bi-phasic growth patterns. Often such phenotypes cannot be predicted but need to be revealed in high-throughput MBR experiments. Furthermore, strain-specific growth patterns may change completely and unpredictably in different nutrition media with, e.g., different carbon sources. Therefore, to fully characterize mutant strain libraries, many high-throughput MBR growth experiments are needed, resulting in big data sets that have to be evaluated accordingly. Thus, researchers are often faced with growth curves displaying a great variety of shapes, which all need to be processed for extracting meaningful performance indicators.

To calculate the growth rate from an individual cultivation, the corresponding data subset covering only the exponential growth phase must be determined. Since in MBR many cultivations are performed in parallel, automation of this data processing task greatly facilitates the experimental evaluation. With typically short microbial cultivation times of 1 day, the generation frequency of such data sets to be evaluated is very high. Most importantly, the task of biological fitness testing of microbial mutant strain libraries should follow a standardized protocol covering both wet-lab experiments and data processing to enable fair comparison. Apparently deviating results, e.g., a significantly higher growth rate of a mutant strain compared to the wild type strain, are ideally discovered in an automated procedure, and then followed by further in-depth manual evaluation including confirmatory growth experiments.

To efficiently handle the massive data load obtained by MBR, scripting languages are ideally suited for rapid development of automated data processing routines, yet a very few with specific features are reported and freely available [5–7]. Here, a MATLAB code is presented that facilitates calculation of growth rates in an automated way based on biomass readings at high temporal resolution from individual cultivations conducted in parallel. This calculation is easily parallelized, as it is demonstrated for all 48 cultivations taking place simultaneously in one cultivation plate in the BioLector [4] MBR device. Growth rate calculation is based on the exponential growth model, $\frac{{dc_{X} }}{dt} = \mu \cdot c_{X}$, considering the growth rate *μ* to be constant. Therefore, cultivation conditions have to be defined in a meaningful way to avoid limited growth originating from insufficient maximum oxygen transfer rates or insufficient pH buffering capacity.

The presented code is applied on an example data set from a *Corynebacterium glutamicum* strain screening campaign and results are compared to literature where data was processed manually. In principle, the code is not restricted to a specific MBR system or scripting language, and thus, can be easily adapted for different microbial cultivation systems or MBR equipment providing biomass data at high temporal resolution [8, 9]. The annotated source code is given as additional material and can be freely downloaded, used and modified without any restriction (Additional file 1).

## Main text

### Specification of MATLAB code

The presented MATLAB code requires five input arguments: (1) a vector of time stamps (2) a vector of corresponding BS readings (= biomass data) blanked by the initial value, (3) a vector of corresponding BS measurement errors, (4) a BS value as user-defined limit of quantification (LOQ) and (5) the adjusted R^2^ that has to be reached for the regression-based growth rate calculation from the data set.

The code, depicted in Codebox 1, is designed to detect the exponential growth phase from a given data set by iteratively calculating a growth rate. From that calculation, several stopping criteria serving as metric for recognition of the exponential growth phase have to be fulfilled. During the first iteration, a time series containing all BS signals between the first measurement where BS exceeds the LOQ and the final measurement is evaluated. Typically, the final measurement is taken during the stationary phase, i.e., after completion of growth. If the stopping criteria (cf. below) are not met, the final measurement is removed for the next iteration, i.e., the penultimate measurement is set as new final measurement.

**Codebox 1:**
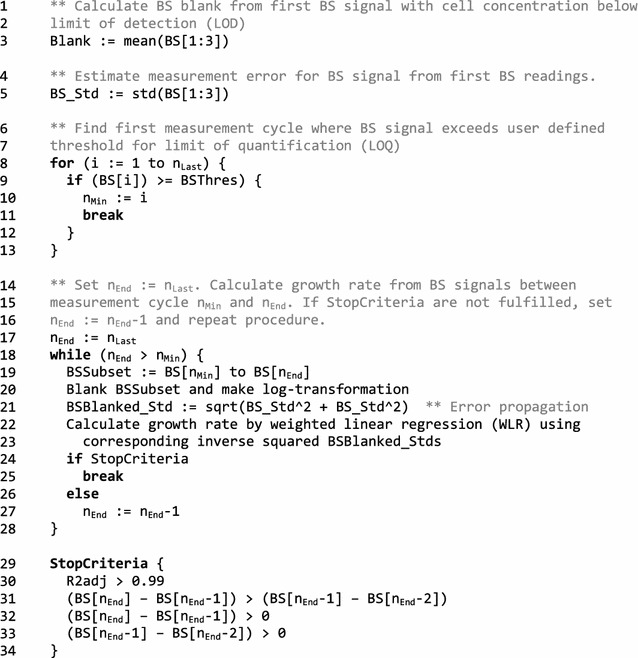
Pseudocode for automated calculation of growth rates, using backscatter (BS) signal as example biomass signal. The procedure starts with the calculation of the blank (zero) value from first BS signals, where the cell concentration is below the limit of detection LOD (lines 1–3). These first BS signals below the LOD are also used to calculate the BS measurement error which is considered to be additive for all BS signals (lines 4, 5). Next, the measurement cycle is identified where the BS signal exceeds the user defined limit of quantification LOQ (lines 6–13). To detect the exponential growth phase from the complete BS data set, a BS subset is extracted ranging from measurement cycles where the BS signal reaches the LOQ to the last one. For this BS subset, the growth rate is calculated by a weighted linear regression and several stopping criteria are evaluated. If these criteria are not fulfilled, a new BS subset is evaluated from which the last measurement is removed. This procedure is repeated until the stopping criteria are fulfilled. Please note: this is implemented as for-loop in the MATLAB function (lines 14–28). Three stopping criteria are defined: A certain adjusted R^2^ from the regression has to be reached. The last biomass increase has to be higher than the previous one, and these two increases need to have a positive sign (lines 29–34)

Three conditions are defined as stopping criteria: First, the adjusted R^2^ from the regression must reach a certain threshold, a value > 0.99 was found to be suitable. This criterion alone is not sufficient, since the data used for regression show a high temporal resolution, so that adjusted R^2^ is still satisfied if several non-wanted data points from the stationary phase are included. Thus, the second stopping criterion is that the increase in biomass in the last measurement cycle needs to be higher than in the previous one. Finally, the third criterion is that these two biomass increases must not be negative, which is sometimes observed as technical measurement artifact during transition from exponential to stationary phase.

The blanked BS readings are transformed by natural logarithm, $\overline{{c_{X} }} = \ln \left( {c_{X} } \right)$, to linearize the growth data for calculating the growth rate according to $\mu = \frac{1}{{c_{X} }} \cdot \frac{{dc_{X} }}{dt} \approx \frac{{\Delta \ln \left( {c_{X} } \right)}}{\Delta t}$. Although non-linear regression (NLR) is considered as “gold-standard”, linear regression (LR) after data transformation results in highly comparable growth rates as discussed below. NLR requires an initial guess since it is an iterative procedure, and this initial guess is reasonably calculated by LR from transformed data. Because each BS signal *c*_*X*_ is connected with a corresponding measurement error $\delta_{{c_{X} }}$, the growth rate calculation is performed as weighted linear regression (WLR). Therefore, errors are transformed accordingly by $\delta_{{\overline{{c_{X} }} }} = \ln \left( {c_{X} } \right)^{{\prime }} \cdot \delta_{{c_{X} }} = \frac{1}{{c_{X} }} \cdot \delta_{{c_{X} }}$ and the inverse squared transformed errors, $\delta_{{\overline{{c_{X} }} }}^{ - 2}$, are used as weights.

### Processing of data from microbial high-throughput MBR cultivations

**Fig. 1:**
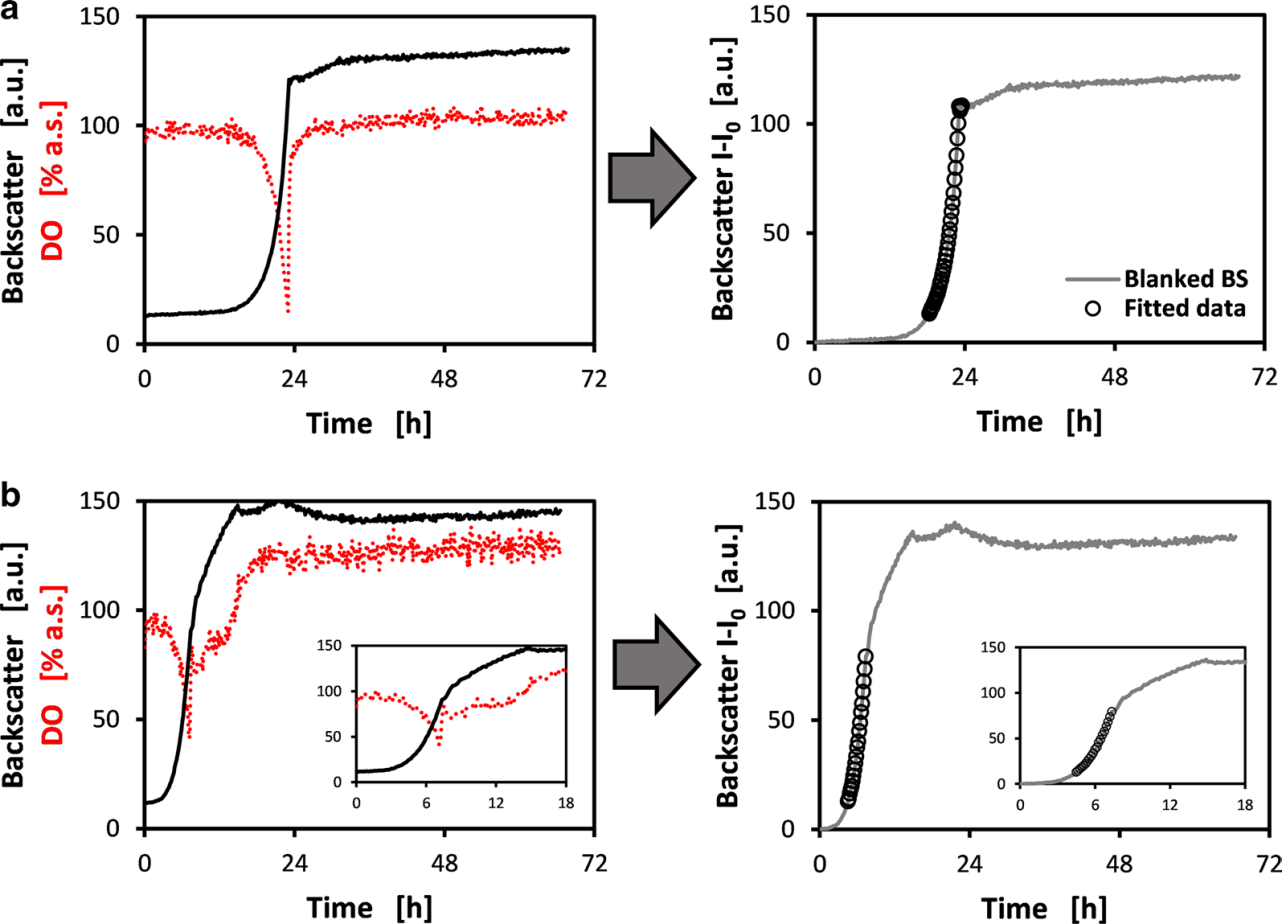
Growth kinetics of *C.* *glutamicum* and *E.* *coli* from BioLector cultivations and depiction of data processing for automated growth rate calculation. **a** Online monitored backscatter and dissolved oxygen (DO) signal for *C. glutamicum* in CgXII medium with 10 g/L glucose, a filling volume of 1000 µL and a shaking frequency of 1000 rpm. The panel on the right shows the processed biomass data, i.e. blanked backscatter and data points determined automatically for calculation of growth rate. Propagated measurement error for blanked BS signal was calculated to 0.39 a.u. **b** Online monitored signals like in part **a**, but for *E. coli* in M9 medium with 20 g/L glucose, a filling volume of 1000 µL and a shaking frequency of 1400 rpm. Propagated measurement error for blanked BS signal was calculated to 0.34 a.u. Right panel analogous to the one in part A. Insets in part B magnify the first 18 h of cultivation. Measurement cycle time for recording backscatter and DO signals was set to 9 min for both *C.* *glutamicum* and *E.* *coli* cultivations

To obtain valid growth rates, cultivation conditions must be applied ensuring that growth is limited by internal factors of the cell only and not by external factors. This is seen for *C. glutamicum* and *Escherichia coli* in the left panel of Fig. 1a and b, respectively. For both growth experiments, conducted in the BioLector MBR, conditions were chosen that result in an exponential growth phase of the cultures. *C.* *glutamicum* was grown in CgXII mineral medium [10] with 10 g/L glucose as carbon source (left panel of Fig. 1a). For the chosen batch mode of operation with 1000 µL cultivation volume and the microplate shaken at a frequency of 1000 rpm, the BS signal increases exponentially from approximately 15 h to 24 h. The dissolved oxygen (DO) signal drops accordingly, showing a sharp rise back to 100% at approximately 24 h, which confirms glucose depletion at this point. Thus, the cultivation conditions are determined to be suitable for *C.* *glutamicum* strain screening and calculation of growth rate. In the case of *E.* *coli*, two growth phases can be derived from the online monitored biomass and DO signals (left panel of Fig. 1b). Here, the mineral medium M9 [11] with 20 g/L glucose as carbon source was used, with a filling volume of 1000 µL and a shaking frequency of 1400 rpm. An exponential increase of biomass signal is seen until approximately 7 h, with the DO signal dropping accordingly until a sharp rise at the same time point. Afterwards, a second, retarded growth phase is visible until approximately 15 h, accompanied by a slowly increasing DO signal. Presumably, excreted acetate and other overflow metabolites from the first exponential growth phase are consumed now, which is a known phenomenon for *E. coli* [12].

After applying the MATLAB code on the data shown in the left panels of Fig. 1, the resulting processed data is depicted in the corresponding right panels. The single exponential growth phase of the *C.* *glutamicum* culture is detected precisely, also for a second replicate cultivation. For *E.* *coli*, exhibiting a first exponential and a second non-exponential growth phase, the MATLAB code is able to detect the first phase safely for all three replicate cultivations, of which one is shown. The growth rate for *C.* *glutamicum* and *E.* *coli* is calculated to 0.46 and 0.61 h^−1^ on average, respectively. In case NLR instead of WLR is used, the same growth rates are determined (cf. Table 1), indicating that WLR of transformed data is a suitable and reliable method for the determination of growth rates (Additional files 1, 2, 3, 4, 5).

**Table 1:** Comparison of regression methods for growth rate calculation using the presented MATLAB code

Cultivated organism	Growth rate with 95% CI from regression method	
	WLR after log-transformation	NLR
*C. glutamicum*		
Replicate #1	0.455 (0.448–0.462)	0.456 (0.448–0.463)
Replicate #2	0.456 (0.448–0.464)	0.457 (0.449–0.465)
*E. coli*		
Replicate #1	0.601 (0.580–0.622)	0.603 (0.582–0.624)
Replicate #2	0.615 (0.598–0.632)	0.616 (0.599–0.633)
Replicate #3	0.617 (0.599–0.635)	0.618 (0.600–0.636)

Growth rates with corresponding 95% confidence interval (CI) are shown for each replicate cultivation of *C. glutamicum* (n = 2) and *E.* *coli* (n = 3)

*WLR* weighted linear regression, *NLR* non-linear regression

### Application example: evaluation of biological fitness of genome reduced *C.* *glutamicum* strains

The presented MATLAB code was used to calculate growth rates from a BioLector raw data set of a previously reported characterization of a library of genome reduced *C.* *glutamicum* strains derived from wildtype ATCC1303 [13] or lysine producer DM1933 [14]. An implementation of this workflow is found as annotated MATLAB Live Script in Additional file 2. In Table 2, the resulting growth rates were compared to those previously reported [13, 14]. More specifically, when comparing the deviation of the automatically calculated growth rates to the data from literature, $Dev.\left[ \% \right] = \left| {1 - \frac{{\mu_{autom.} }}{{\mu_{Lit.} }}} \right| \cdot 100$, the following is found: In most cases (13 out of 23) a deviation of 10% or less is seen, few values (8 out of 23) deviated by 15% or less, and only two growth rates show a higher deviation of 16 and 21%. Consequently, growth rates calculated from automated detection of the corresponding growth phase agrees very well with literature, where manual effort was required, e.g., to remove backscatter readings form the stationary phase before data fitting.

**Table 2:** Automated calculation of growth rates from different genome reduced *C. glutamicum* strains with comparison to literature

Strain	Growth rate [h^−1^]	
	Literature	This study (95% CI)
DM1933	0.32 ± 0.01	0.30 (0.29–0.30)
DM1933 ΔCGP1	0.30 ± 0.03	0.30 (0.29–0.31)
DM1933 ΔCGP2	0.31 ± 0.03	0.32 (0.31–0.32)
DM1933 ΔCGP3	0.31 ± 0.02	0.32 (0.31–0.32)
DM1933 ΔCGP123	0.33 ± 0.03	0.33 (0.32–0.34)
GRLP16	0.31 ± 0.02	0.35 (0.34–0.35)
GRLP23	0.33 ± 0.02	0.31 (0.30–0.32)
GRLP41	0.31 ± 0.01	0.29 (0.28–0.30)
GRLP42	0.36 ± 0.06	0.30 (0.29–0.31)
GRLP46	0.30 ± 0.04	0.32 (0.32–0.33)
GRS37	0.45 ± 0.03	0.51 (0.51–0.52)
GRS40	0.44 ± 0.03	0.50 (0.49–0.51)
GRS41	0.41 ± 0.04	0.47 (0.46–0.47)
GRS42	0.39 ± 0.01	0.47 (0.46–0.48)
GRS46	0.43 ± 0.03	0.50 (0.49–0.50)
GRS45	0.31 ± 0.02	0.34 (0.34–0.34)
GRS47	0.41 ± 0.03	0.48 (0.47–0.49)
GRS48	0.45 ± 0.01	0.49 (0.49–0.50)
GRS53	0.44 ± 0.03	0.47 (0.47–0.48)
MB001	0.43 ± 0.04	0.48 (0.47–0.48)
GRS16	0.44 ± 0.03	0.49 (0.48–0.50)
GRS23	0.46 ± 0.02	0.47 (0.47–0.48)
WT^a^	0.43 ± 0.04	0.46 (0.45–0.46)
		0.46 (0.45–0.46)

Data originates from a BioLector raw data set and results are compared to previously reported data collections [13, 14]

Data from literature is given as mean ± standard deviation. Strain order corresponds to Additional file 2

*CI* confidence interval

^a^For strain WT two data sets from growth duplicates were processed

Furthermore, the presented code was shown to reproducibly detect the exponential phase from multi-phasic growth patterns (cf. Fig. 1b). It can be used to rapidly characterize mutant strains for different growth regimes, such as different media compositions that allow a differential analysis of genome modification impact on biological fitness based on growth rate [13].

## Conclusions and outlook

A MATLAB code was presented that allows for model-based automated calculation of growth rates from high-throughput parallelized microbial cultivations that were monitored at a high-temporal resolution. The code uses online biomass data (here: blanked backscatter signal) and weighted linear regression in an iterative procedure for reliable identification of exponential growth phase with growth rate calculation. By applying cultivation conditions that satisfy the assumptions of the underlying model of exponential growth, the code demonstrated to safely detect the exponential growth phase and reproducible growth rate calculation, even in bi-phasic microbial cultivations. An application example data set from biological fitness assessment of *C. glutamicum* genome reduced strains was processed using the presented code and growth rates were in very good agreement with previous results. It is reasonable to assume, that the presented code can be also applied to different growth conditions or changed media compositions, if the obtained data still match to the exponential growth model.

The use of MBR systems in high-throughput mutant strain screening campaigns and accelerated microbial bioprocess development easily produces a huge amount of data. Consequently, resulting data needs to be processed in an automated, standardized and efficient way. Using standardized data output from MBR in spreadsheet formats, MATLAB routines can be implemented which facilitate elevated experimental throughput. Most importantly, the application of standardized growth rate calculation methods enables a fair comparison of screened strains regarding biological fitness.

## Limitations

Biomass monitoring in the BioLector MBR relies on BS measurements, which is the key feature to temporal high-density data acquisition in microplate cultivation [4, 15]. In contrast to the determination of biomass concentration by cell dry weight, optical measurement for biomass determination via BS was found to be organism specific [16]. Hence strain specific correlations for BS and optical density measurements need to be determined and have been reported [8]. Issues like optical crosstalk or cell morphology have to be considered for biomass determination by optical measurements in general [9, 17, 18]. Consequently, for meaningful interpretation of screening results, biomass calibration should be an integral part of strain screening campaigns with the BioLector device and other MBRs. Furthermore, cultivation conditions (media composition, filling volume, shaking frequency) should be verified to fulfill the underlying assumptions of the exponential growth model since the presented MATLAB code calculates growth rates based on this model. Therefore, conditions causing diauxic growth behavior, e.g., oxygen limitation or the use of complex media, may result in erroneous growth rate calculation. In such cases, a differential method that calculates a dynamic growth rate over time may be more suitable for data interpretation for changing growth regimes. This holds especially true for microscale cultivations of microorganisms that do not show an exponential growth [19] or exhibit complex morphology [20].

## Additional files

**Additional file 1.** MATLAB file for automated calculation of growth rates from BioLector backscatter time series based on weighted linear regression.

**Additional file 2.** MATLAB live script executing all steps for automated growth rate calculation as annotated working example.

**Additional file 3.** MATLAB file for automated calculation of growth by using non-linear regression, needed for the MATLAB live script working example.

**Additional file 4.** Spreadsheet file containing BioLector raw data set with backscatter and DO readings for twenty-four cultivations of *C.* *glutamicum* strains, used for calculation of growth rates in the application example.

**Additional file 5.** Spreadsheet file containing BioLector raw data set with backscatter and DO readings for three replicate cultivations of *E.* *coli.*

## Abbreviations

a.u.: arbitrary units
BS: backscatter
DO: dissolved oxygen
LOD: limit of detection
LOQ: limit of quantification
LR: linear regression
MBR: microbioreactor
NLR: non-linear regression
WLR: weighted linear regression
